# Automatic Detection of Arrhythmia Based on Multi-Resolution Representation of ECG Signal

**DOI:** 10.3390/s20061579

**Published:** 2020-03-12

**Authors:** Dongqi Wang, Qinghua Meng, Dongming Chen, Hupo Zhang, Lisheng Xu

**Affiliations:** 1 Software College, Northeastern University, Shenyang 110169, China; wangdq@swc.neu.edu.cn (D.W.); 1871111@stu.neu.edu.cn (Q.M.); zhanghp@stumail.neu.edu.cn (H.Z.); 2 College of Medicine and Biological Information Engineering, Northeastern University, Shenyang 110169, China; xuls@bmie.neu.edu.cn

**Keywords:** arrhythmia detection, ECG, multi-resolution representation, deep learning

## Abstract

Automatic detection of arrhythmia is of great significance for early prevention and diagnosis of cardiovascular disease. Traditional feature engineering methods based on expert knowledge lack multidimensional and multi-view information abstraction and data representation ability, so the traditional research on pattern recognition of arrhythmia detection cannot achieve satisfactory results. Recently, with the increase of deep learning technology, automatic feature extraction of ECG data based on deep neural networks has been widely discussed. In order to utilize the complementary strength between different schemes, in this paper, we propose an arrhythmia detection method based on the multi-resolution representation (MRR) of ECG signals. This method utilizes four different up to date deep neural networks as four channel models for ECG vector representations learning. The deep learning based representations, together with hand-crafted features of ECG, forms the MRR, which is the input of the downstream classification strategy. The experimental results of big ECG dataset multi-label classification confirm that the F1 score of the proposed method is 0.9238, which is 1.31%, 0.62%, 1.18% and 0.6% higher than that of each channel model. From the perspective of architecture, this proposed method is highly scalable and can be employed as an example for arrhythmia recognition.

## 1. Introduction

Cardiac arrhythmia is an essential manifestation of cardiovascular disease (CVD). As the most significant cause of death in the world, CVD causes about 17.9 million deaths every year, accounting for 31% of the total global deaths [1]. Electrocardiogram (ECG) is a comprehensive tool widely used by clinicians in hospitals which captures the propagation of electrical signals in the heart from the body surface and then the state of the cardiovascular system can be detected through the morphology and rhythm of ECG. Because of its non-invasive and easily accessible characteristics, ECG has played a vital role in the diagnosis of various arrhythmia and acute coronary syndromes [2,3].

Automatic Detection of Arrhythmia (ADA) refers to the use of computer equipment to replace the tedious and time-consuming manual ECG data analysis, which will not lead to recognition problems due to expert differences or fatigue. The ADA problem has been widely researched in recent decades and the research can be generally divided into the following three steps: ECG data preprocessing, feature extraction, and classification, where data preprocessing includes noise filtering, baseline removal, and signal segmentation [4]. A typical solution for ADA is, similarly to [5], for each heartbeat, to combine the features extracted by wavelet transformation (WT) and independent component analysis (ICA) with the four RR interval features designed and then transmit all of them to a support vector machine (SVM) for classification. Acharya et al. extracted 13 types of non-linear features for a heartbeat signal and used the decision tree (DT) method to identify four beats of Normal, Atrial fibrillation, Atrial flutter and Ventricular fibrillation [6]. In [7], the authors made use of recurrence quantification analysis (RQA) technology to extract the hidden nonlinear features of ECG signals and feed these features to a rotation forest (ROF) classifier. Although these traditional methods claim that they achieved satisfying recognition performance, it is evident that this type of automatic detection solution heavily relies on the use of expertise for feature extraction or feature selection, which is not only difficult to generalize, but the performance is also not good enough for practice usage.

In recent years, with the rapid development of deep learning, this technology has dramatically improved the state of the art in image classification, target detection, speech recognition, and other fields [8,9,10,11,12]. Deep learning models can recognize patterns from the input data and learn useful features without a large amount of data preprocessing and feature engineering [13], which is also suitable for ECG data mining. Reference [14] used a Convolutional Neural Network (CNN) to detect the 2 seconds and 5 seconds ECG segments and achieved a good recognition accuracy. Researchers from Stanford University designed a thirty-four-layer CNN, which can recognize 14 types of arrhythmias and achieved expert performance [15]. Reference [16] proposed an end-to-end depth model to identify 17 types of arrhythmias and claimed it as one of the best multi classification results on the 1000 ECG signal fragments from the MIT - BIH Arrhythmia database for one lead (MLII) from 45 persons so far.

For a deep neural network, model training is a process of a layer-by-layer evolution of the abstraction level of the input data, which means that the outputs of different node layers of the same deep model provide different abstraction level vector representations of the input. At the same time, different deep neural networks try to extract meaningful representations of the input data from different angles. Golrizkhatami et al. exploited multi-stage learned features from a trained CNN and hand-crafted features extracted using well known algorithms to classify heartbeats into five different AAMI class types [17]. In [18], the researchers combined CNN and bi-directional Long Short-Term Memory (LSTM) into a network to achieve the fusion of the two network extraction features. However, the drawback is that the scalability of the model is poor, and its training time will increase exponentially with the number and complexity of the models used. In the field of natural language processing, Tolgahan Cakaloglu and Xiaowei Xu proposed a new method to generate multi-resolution word embedding and proved the effectiveness of the proposed approach through experiments [19]. Inspired by [19], in this paper, we propose a new ECG data representation construction method for the ADA tasks and call the representation constructed by this method the multi-resolution ECG data representation. The proposed method learns multiple features of the input ECG data by using distinct deep neural network models (called the channel models) separately and combining these features through a feature fusion strategy. The channel models used were GoogLeNet, ResNet, SeInceptionNet and SeResNet [20,21,22,23]. Among them, the latter two models are based on the InceptionNet and ResNet with the Squeeze-and-Excitation Network (SeNet) module added [23]. Then, the multi-resolution representation was be transmitted to a gradient boosting algorithm to perform ADA classifier training. For the training, the k-fold cross-validation strategy was applied, and a voting-based model fusion was used to predict arrhythmia. This method not only makes full use of the representation learning ability of channel models but also introduces the expert information in feature engineering, so it achieved a high recognition performance in our experiment.

The main contributions of this work are as follows:

(1) An arrhythmia recognition method based on a Multi-Resolution Representation (MRR) of the ECG signal was proposed. Theoretically, the MRR mechanism can combine all possible features generated by any means. These features can vector representations learned by deep neural networks, categorize information of inputs or features extracted using domain knowledge.
(2) The Convolutional Neural Network’s power of ECG signal representation by the Squeeze-and-Excitation mechanism was strengthened. The SE mechanism models the interdependencies between channels during training and pays more attention to the channel information useful for the final prediction.

As we know, for the reason of patients’ privacy protection, ECG data collection is a tough job, current research works usually use open-source datasets such as MIT-BIH Arrhythmia Database [24], which is limited by the amount of data. In this study, we conducted our research on a very recent bigger ECG dataset from Alibaba Tianchi Cloud Competition, which has 20036 8-lead ECG records. The dataset contains 34 arrhythmia categories, and the task is multi-label classification, which is more challenging.

The rest of this paper is organized as follows. Section 2 introduces the proposed model. Then, some details on the experiment settings are described in Section 3. In Section 4, the experimental results are presented and discussed. Finally, Section 5 concludes the paper.

## 2. Proposed Method

### 2.1. Model Architecture

**Figure 1:**
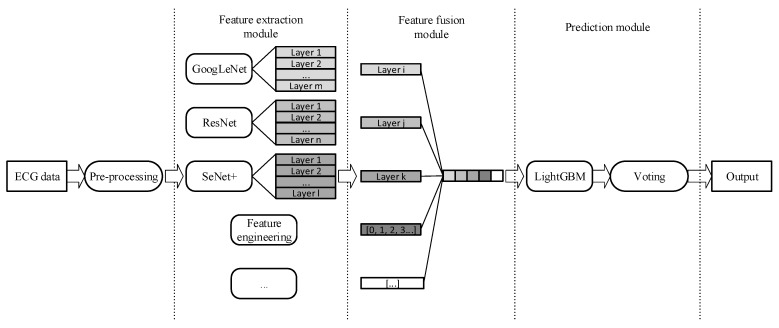
Proposed arrhythmia detection framework based on multi-resolution representation (MRR) of an electrocardiogram (ECG).

The proposed model, illustrated in Figure 1, is composed of the feature extraction module, the feature fusion module, and the prediction module. Firstly, in the preprocessing operation, we applied db6 wavelet transformation [25] to all ECG signals to reduce the impact of noise. Then, in the feature extraction module, the commonly used models in the field of deep learning, GoogleNet, ResNet, SeInceptionNet, and SeResNet, were selected to learn the deep vector representation of ECG data. Moreover, we also fed age, gender, and Heart Rate Variability (HRV) information [26] to the follow-up feature fusion module. Suppose that in the feature extraction module, each layer of the selected and trained deep model provides an abstract understanding (representation) of ECG data. Then, in the feature fusion step, we cose appropriate ones from these understandings and concatenate them with domain knowledge-based features to form a new representation. This new representation is a multi-level abstraction understanding of the ECG signal, and we named it the MRR of ECG. After this, we fed the MRR to a LightGBM model [27]. LightGBM is a gradient boosting framework that uses a decision tree based on the histogram. We expect to use LightGBM to achieve fast running speed and high classification accuracy at the same time. The last part of our proposed method is a model fusion-based classification decision module in which we used a voting method to fuse the models [28]. Researchers interested in re-implementing our proposed method can choose a more complex fusion strategy according to their own needs.

### 2.2. Deep Feature Extraction

#### 2.2.1. Problem Formulation

As described above, the MRR of the ECG signal is constructed on the base of the multi-channel deep models’ training. Each model training itself is a multi-label classification task of input 8-lead ECG records; the output of the trained model is a sequence of probabilities that the input sample belongs to corresponding categories. We use binary cross-entropy to measure training loss; as is shown in Formula 1, the loss function minimizes the cross-entropy between ground truth and the channel model output. x and y denote the deep model output and the ground-truth labels of the input sample, respectively. c is the number of arrhythmia categories in datasets and 1≤i≤c. The loss value is the weighted average of the loss on each category.

(1) $$loss\left(x,y\right)=mean\left(\sum_{i=1}^{c}-w_{i}\left(y_{i}\times \log x_{i}+\left(1-y_{i}\right)\times \log\left(1-x_{i}\right)\right)\right),$$

Considering the uneven distribution of different arrhythmia categories in the datasets, we assigned a weight $w_{i}=1/\log n_{i}$ to each type of arrhythmia to avoid a biased model where $n_{i}$ is the number of corresponding arrhythmia type samples in datasets. In this way, we assign more significant weights to arrhythmia types with a small number of samples to punish the model with a more considerable loss value. Therefore, the model pays more attention to the arrhythmia types with a smaller numbers of samples.

#### 2.2.2. GoogLeNet

**Figure 2:**
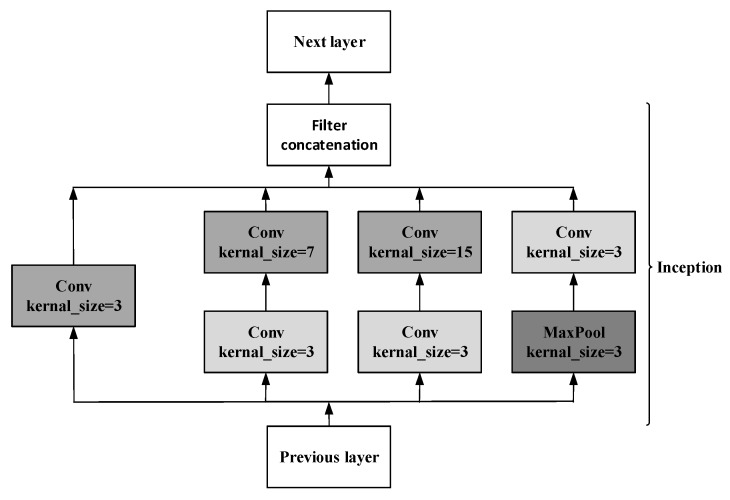
Inception module structure.

The traditional way to improve network performance by increasing the depth and width not only requires more parameters and computing resources but is also prone to overfitting. For this, GoogLeNet did not choose a deeper network but designed an Inception structure to improve the network performance. Referring to the model used in [20], we adjusted the Inception module to adapt to ECG data. As shown in Figure 2, the Inception module has convolution kernels of different sizes, which can extract ECG features of different scales. More abundant features are helpful for more accurate classification.

The whole network consists of 22 layers, including nine inception modules. Global average pooling was used to replace the fully connected layer. Averaging the entire feature map will reduce the calculation amount and increases the training speed and performance at the same time.

In addition, in order to prevent the gradient from disappearing due to depth in forwarding propagation, we employed two auxiliary branches in layer 10 and layer 16 of the model, which can take the output of the middle layer as the result of the classification. As shown in Formula 2, during training, three parts decide the final loss function where Loss2 is the loss of the final output result, and two branches calculate Loss1 and Loss0. The network parameters and structure can be found in the Supplementary Materials.

(2) Loss=Loss2+0.3∗Loss1+0.3∗Loss0,

#### 2.2.3. ResNet

**Figure 3:**
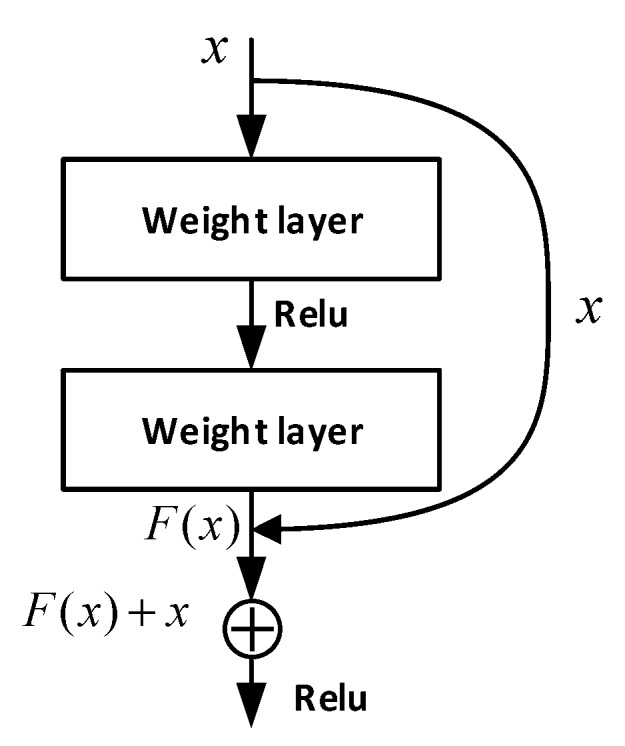
Residual module structure.

ResNet solves the problem of deep network degradation by introducing the deep residual learning framework [21]. As shown in Figure 3, x comes from the upper layer network and F(x) is the output through two-layer neural network. Generally, features from shallow layers are usually lower semantic-level features with a higher resolution. In contrast, representations from deep layers are often more upper semantic-level features with a lower resolution. The combination of features with different resolutions is realized by adding the F(x) and x.

According to the characteristics of ECG data, we modified the original ResNet to achieve a better recognition performance. The network parameters can be found in the Supplementary Materials.

#### 2.2.4. SeNet

**Figure 4:**
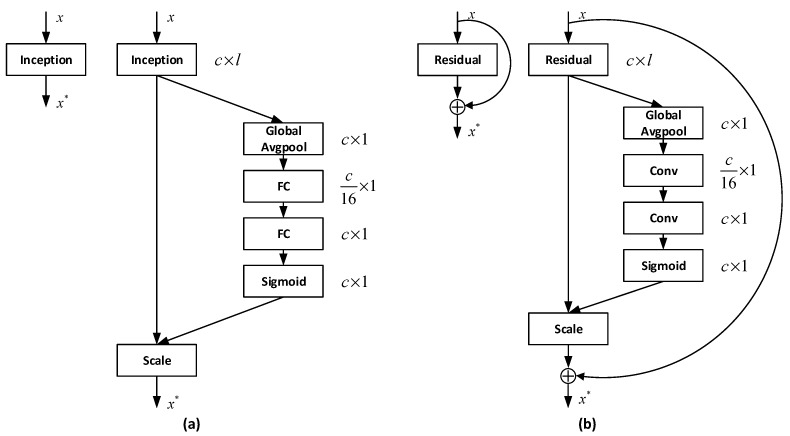
The two types of SeNet module structures: (**a**) SeNet realized by fully connected neurons; (**b**) SeNet realized by convolution operations.

Many existing deep-learning-based arrhythmia detection solutions employ CNN to extract spatial information and do not consider the correlations between different feature channels during training. As is shown in Figure 4, to enhance the role of channel relationship in feature generation, we used two types of SeNet structures in the experiment, where x is the input, $x^{*}$ is the output, and c represents the number of channels. One SeNet structure (a, Figure 4) models the interdependencies between feature channels through fully connected neurons (FC) and the other SeNet structure (b, Figure 4) replaces the FCs with convolution operations (Conv). Both SeNet structures compress the number of convolution channels 16 times and then expand it to the original size. These structures explicitly model the relationship between feature channels and use the feature recalibration strategy to obtain the importance of each feature channel through learning automatically. This strategy has been proven to be able to ‘selectively enhance useful features and suppress less useful features’ in image classification applications [23], which we believe will also be able to serve our ECG signal feature extraction task.

### 2.3. Feature Fusion and Prediction

In addition to the features extracted by the above models, hand-crafted features were also used to construct MRR. In the feature fusion module (see Figure 1), the features extracted by deep neural networks concatenate with nine HRV indexes, age and gender information to be the Multi-Resolution Representation of ECG signal. HRV measures the change of continuous heart rate cycle, which is a valuable index to predict sudden cardiac death and arrhythmia [26]. In this experiment, we extracted nine HRV indexes from the lead II ECG signals, including RR interval standard deviation, maximum RR interval, minimum RR interval, average RR interval, pNN50 (ratio of adjacent RR interval difference higher than 50ms), R wave density, RMSSD (root mean square of adjacent RR interval difference), sampling entropy of RR interval (measuring RR interval change disorder, returning two values). Compared to representation learned by the single deep model, this representation contains the knowledge used in these channel models, so using this representation as input to train downstream classifier will achieve a higher recognition performance.

As shown in Figure 1, we used a LightGBM classifier in the prediction module. The output Multi-Resolution Representation of the feature fusion operation was used as the input of the classifier. LightGBM is a gradient lifting framework based on a decision tree algorithm. It uses the leaf-wise growth strategy to select the node with the most significant gain from the current leaf node each time to split. The model can effectively prevent overfitting by controlling the depth of the tree and the number of leaf nodes [27]. Finally, k models obtained by the k-fold method were used to predict the test set and the final classification results were determined by voting. A voting scheme is a standard model fusion scheme which is simple, fast, and easy to operate.

## 3. Experiments

### 3.1. Data Sets

The experimental data used are from the “Hefei high tech Cup” ECG Human-Machine Intelligence Competition held by the Tianchi platform [29,30,31]. This dataset contains 20,036 8-lead ECG records (including lead I, II, V1, V2, V3, V4, V5 and V6), each of which has a length of 10 s and a total of 34 categories. The sampling frequency was 500 Hz and the unit voltage was 4.88 mv. Moreover, each record also contains the patient’s age and gender information. These records were provided by the Engineering Research Center of the Ministry of Education of Hangzhou Normal University Mobile Health Management System. Table 1 gives details on this dataset.

### 3.2. Data Augmentation

As shown in Table 1, although there are more than 20,000 records, the dataset contains more than 30 types of arrhythmia, and the number of samples in many categories is relatively small. Therefore, we used three data enhancement schemes in the experiment to avoid overfitting.

(1) Randomly select a scaling factor τ from the normal distribution (0, 0.1), and then use (1+τ) to scale the original signal. This method can simulate groups of different constitutions, because people of different constitutions tend to have various heart rhythm fluctuations.
(2) Translation to generate more data is a common data augmentation method in the image field [32]. Inspired by this method, we translated the ECG signal as a whole to simulate the same individual’s ECG collected at different times.
(3) During training, use an all-zero segment of (0, 1) s length fragment to randomly mask the ECG signal to prevent the model from focusing too much on some local features. [33]

### 3.3. Experimental Setting

We trained and tested our model on a server with 32 Cores Intel Xeon E5-2620 CPU, 128GB memory, and two GeForce RTX 2080 Ti cards. This server runs a Linux 3.10.0 system (Cloudhin, Shanghai, China). The development language we chose was Python and the deep learning framework was PyTorch 1.0.1.

We selected sigmoid function as the activation functions and binary cross-entropy as the loss function when training the deep learning models. The entire model was trained using the Adam optimizer with an initial learning rate of 0.001. In a total of 50 epochs of training, the learning rate was be multiplied by 0.1 when the number of epochs reached 35 and 45. In addition, we put a Batch Normalization layer [34] following each convolutional layer to accelerate the model’s convergence. Dropout was also introduced to prevent overfitting [35]. For further parameters-setting information, please see the Supplementary Materials.

### 3.4. Evaluation Metrics

In order to evaluate the performance of the proposed method, we employ typical classification metrics to measure experimental results. As is shown in formula 3–5, these metrics include Precision, Recall and F1 score.

(3) $$P=\frac{TP}{TP+FP},$$

(4) $$R=\frac{TP}{TP+FN},$$

(5) $$F1=\frac{2\times P\times R}{P+R},$$

where P and R denote the Precision and Recall, respectively. TP represents the number of samples correctly classified in a certain category. FP is the number of samples not belonging to a certain category but which are classified as this category. FN represents the number of samples mistakenly classified into other categories in a certain category.

## 4. Results and Discussion

### 4.1. Results

The experiment was carried out by 5-fold cross-validation. Without the help of other data, we use 20% of the data as the test set each time and average the results of all folds. Except for the test set, we divided the rest of the data into a training set and a verification set according to the method of cross-validation and selected the best sub-model through verification. The features extracted by channel models are middle layer features generated by the last fully connected layer of the corresponding model. Meanwhile, it should be noted that since the experiment involves two steps of training (the channel model training and the LightGBM classifier training), in order to ensure the performance of the experiment, the data set division of the two steps was consistent.

Table 2 lists the experimental results of several schemes under cross-validation. Column 1 indicates the methodology and column 2–4 indicate the performance of 34 types of arrhythmia classifiers, including F1 score, Precision, and Recall. In order to facilitate experimental comparison, LightGBM was still selected to train hand-crafted features. By comparing the F1 score and Recall value, we can find that the performance of the proposed method is better than the channel models, with an F1 score and a recall rate of 0.9238 and 0.9107, respectively. However, we found that the opposite situation occurred in the Precision. The method with a lower F1 score has a higher precision rate, which may be caused by the higher missed diagnosis rate of the model, and the lower true positive and false positive.

### 4.2. Discussion

As we can see, the feature extraction module (see Figure 1) can be easily extended by adding more deep learning models into it. To further illustrate the effectiveness of this Multi-Resolution Representation construction strategy, we used the following two schemes for each channel model.

(1) For a specific channel model, a sub-model will be obtained in each fold’s training of a 5-fold cross validation. These sub-models vote to decide the prediction result on the test set.
(2) For a specific channel model, differently from the strategy of using multiple models to construct MRR in the proposed scheme above, we used the five sub-models obtained by cross validation to extract features from the dataset and combine them as a new MRR of ECG data. Then feed the new MRR to LightGBM for training who vote to decide the prediction result on the test set.

In Table 3, scheme_1 and scheme_2 are the two schemes compared. The F1 score and recall rate shows that in each channel model, the results obtained by the scheme based on MRR are better than direct voting. This shows that the multi-resolution representation contains more information than the final prediction results, so the model fusion using the multi-resolution representation has a better recognition performance than using the prediction results directly. Furthermore, compared with the proposed method in Table 2, the experimental results of the multi-resolution representation of multiple models are better, which proves the effectiveness of the proposed scheme.

Moreover, we find that SeResNet performs better than ResNet, which verifies our previous point of view: considering the relationship between feature channels helps the model learn more information so that it can improve the model’s ability to classify ECGs.

Table 4 compares the number of parameters of a ResNet with and without the two kinds of SeNet structures. Compared to the original ResNet, the SeNet enhanced network only adds 0.41–6.22% additional parameters, which is a good performance improvement.

The main advantages of our proposed method are as follows:

(1) It has good scalability and can fuse the useful information in any recognition scheme. Compared with the method used in [18], the training time of our model only has linear growth, which mainly depends on the time consumption of each fusion scheme.
(2) The proposed method can combine the advantages of each recognition scheme. Because the classification method used in MRR generation filters the features according to the information gain of the nodes, the method is more robust than the ones that used the prediction results directly for model integration. In the worst case, the results will not be worse than every single model. On this basis, the scheme of model integration can further improve accuracy.

## 5. Conclusions

In this paper, we propose a multi-resolution representation-based arrhythmia detection solution. This solution integrates vector representations learned by different popular deep neural networks and commonly used hand-crafted ECG features to be the new representation for downstream ECG classifier. We call the integrated representation the ’Multi-Resolution Representation’ because it reveals the different abstraction level information of ECG signals. The proposed method has the following advantages: (1) High scalability. The MRR construction strategy can be extended to incorporate existing deep models into its framework as its channel models. (2) Stable performance. Theoretically, in the worst case, this method will still be able to achieve as good a performance as good as channel model-based solutions.

We carried out the arrhythmia detection experiment on a large dataset with 20,036 8-lead medical ECG records and 34 arrhythmia categories. The experimental results show that the proposed method achieved a high recognition performance. The F1 score was 0.9238, which is 0.883% higher than the channel models used on average.

## Figures and Tables

**Table 1:** Statistics of dataset used in this study.

#	Type	Records
1	Low QRS voltages	3
2	Right axis deviation	1124
3	Paced rhythm	16
4	T wave change	3479
5	Left axis deviation	1124
6	Atrial fibrillation	120
7	Nonspecific ST segment anomaly	64
8	Abnormal Q-wave in inferior wall	52
9	Poor R wave progression of the front wall	16
10	ST segment change	286
11	First degree atrioventricular block	142
12	Left bundle branch block	25
13	Right bundle branch block	551
14	Complete left bundle branch block	25
15	Left anterior fascicular block	35
16	Right atrial enlargement	32
17	Short PR interval	23
18	Left ventricular high voltage	414
19	Sinus bradycardia	5264
20	Early repolarization	22
21	Normal sinus rhythm	9501
22	Fusion beat	7
23	ST-T change	299
24	Nonspecific ST segment and T wave anomaly	16
25	Rapid ventricular rate	29
26	Nonspecific T wave anomaly	34
27	Ventricular premature beat	543
28	Atrial premature beat	314
29	Sinus arrhythmia	901
30	Complete right bundle branch block	418
31	Sinus tachycardia	4895
32	Incomplete right bundle branch block	126
33	Clockwise rotation	35
34	Counterclockwise rotation	60
-	Total	29995

**Table 2:** Classification performance for each channel model and the proposed method.

Type	F1 Score	Precision	Recall
GoogleNet	0.9118	0.9398	0.8854
SeInceptionNet	0.9181	0.9480	0.8902
ResNet	0.9130	0.9399	0.8876
SeResNet	0.9183	0.9427	0.8951
Hand-crafted fea	0.7922	0.9051	0.7045
Multi-Resolution	0.9238	0.9372	0.9107

**Table 3:** Comparison of two experimental schemes in each single model.

Type	F1 Score		Precision		Recall	
	scheme_1	scheme_2	scheme_1	scheme_2	scheme_1	scheme_2
GoogleNet	0.9118	0.9173	0.9398	0.9305	0.8854	0.9044
SeInceptionNet	0.9181	0.9196	0.9480	0.9320	0.8902	0.9077
ResNet	0.9130	0.9190	0.9399	0.9331	0.8876	0.9055
SeResNet	0.9183	0.9205	0.9427	0.9342	0.8951	0.9072

**Table 4:** Number of parameters of the ResNet with and without SeNet.

Net Type	Parameters
ResNet	37899938
SeResNet_1	38057122
SeResNet_2	40257698

## Supplementary Materials

The following are available online at https://www.mdpi.com/1424-8220/20/6/1579/s1.
