# Supplementary figures and images for: Automatic Detection of Arrhythmia Based on Multi-Resolution Representation of ECG Signal
Source: Sensors (Basel). 2020 Mar 12;20(6):1579. doi: 10.3390/s20061579 (PMC7175329; doi:10.3390/s20061579)

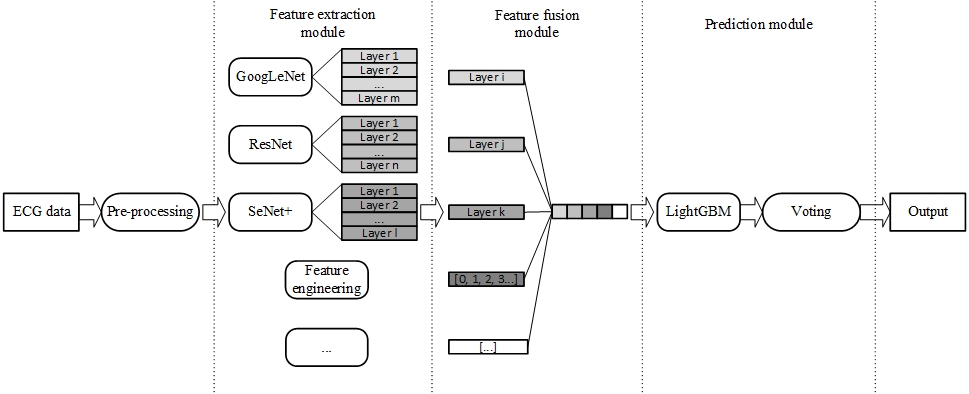

Supplement: Supplementary file 1 [file sensors-20-01579-s001.zip › Supplementary Materials/Figure/Figure 1.jpg]

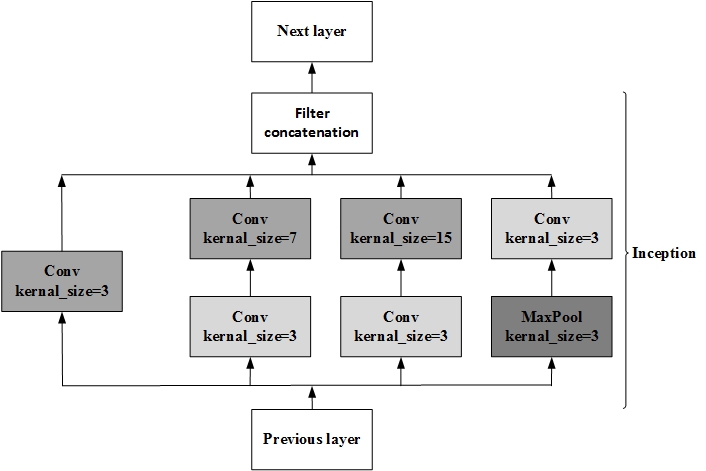

Supplement: Supplementary file 1 [file sensors-20-01579-s001.zip › Supplementary Materials/Figure/Figure 2.jpg]

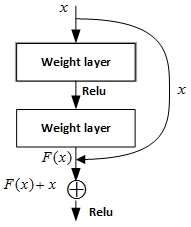

Supplement: Supplementary file 1 [file sensors-20-01579-s001.zip › Supplementary Materials/Figure/Figure 3.jpg]

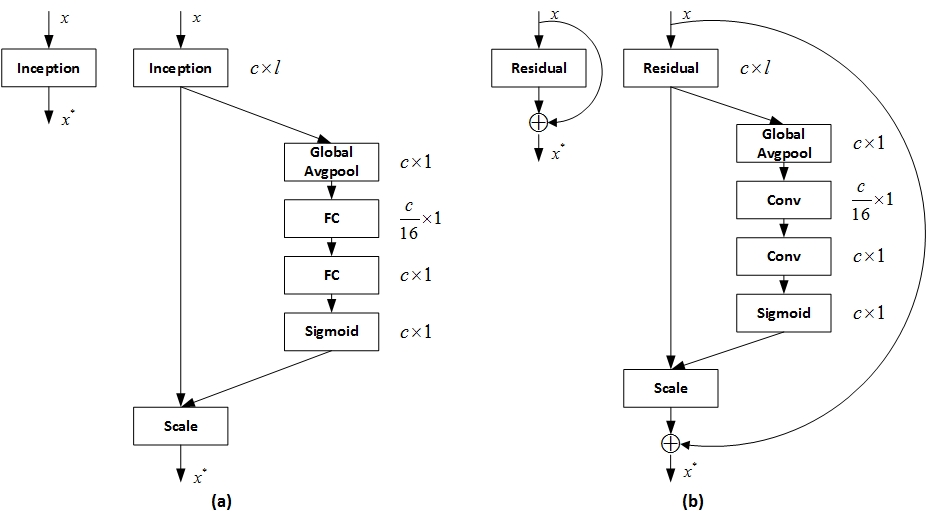

Supplement: Supplementary file 1 [file sensors-20-01579-s001.zip › Supplementary Materials/Figure/Figure 4.jpg]

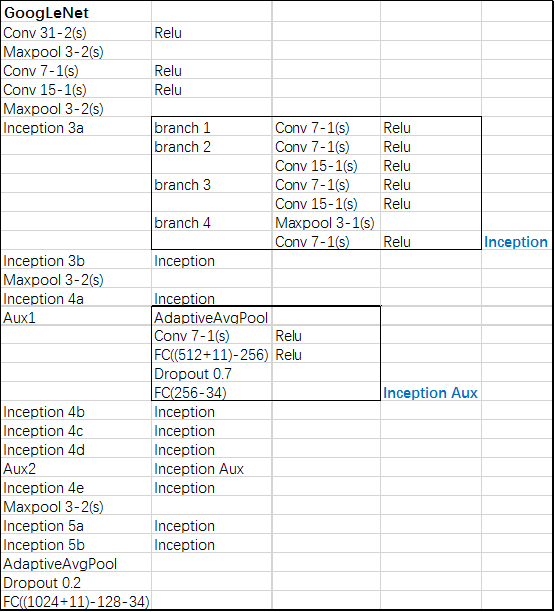

Supplement: Supplementary file 1 [file sensors-20-01579-s001.zip › Supplementary Materials/Structure and parameters/googLeNet.jpg]

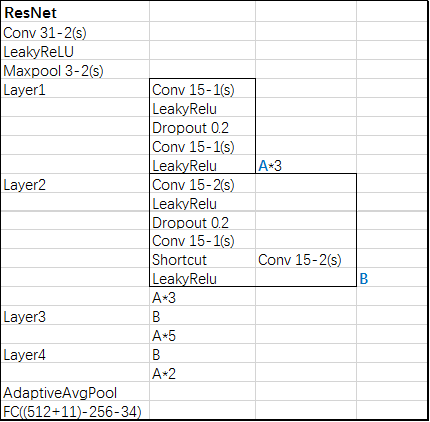

Supplement: Supplementary file 1 [file sensors-20-01579-s001.zip › Supplementary Materials/Structure and parameters/resNet.jpg]

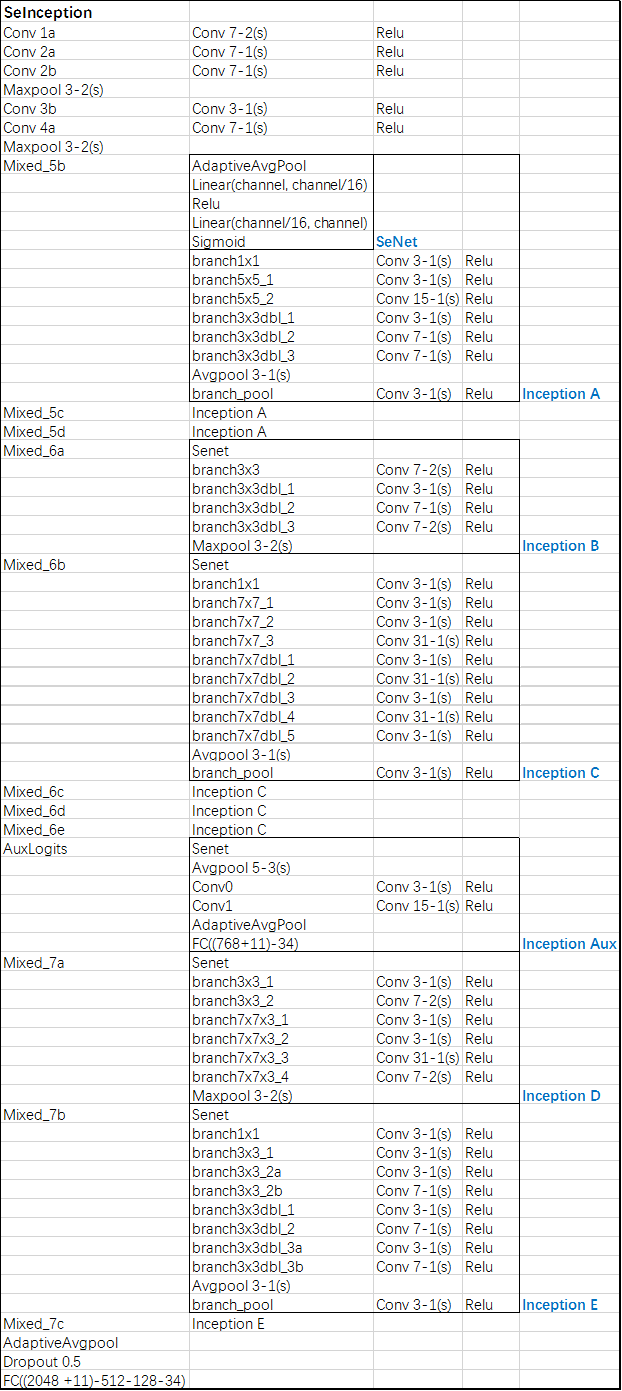

Supplement: Supplementary file 1 [file sensors-20-01579-s001.zip › Supplementary Materials/Structure and parameters/seInceptionNet.jpg]

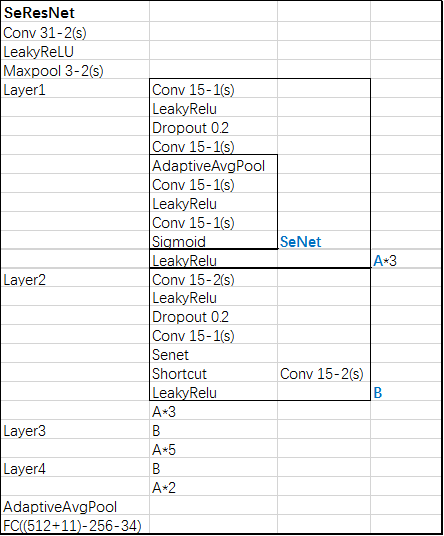

Supplement: Supplementary file 1 [file sensors-20-01579-s001.zip › Supplementary Materials/Structure and parameters/seResNet.jpg]
